# Supplementary material for: Asymmetric Synthesis of Quaternary Hydantoins via a Palladium-Catalyzed Aza-Heck Cyclization
Source: J Am Chem Soc. 2025 Nov 14;147(49):44692–8. doi: 10.1021/jacs.5c16022 (PMC12703750; doi:10.1021/jacs.5c16022)
Supplement: Supplementary file 2 [file ja5c16022_si_002.zip › All NMR FID Files/S3/S3_AllNMR/2/pdata/1/email_EM04-133-crd_2_1.pdf]

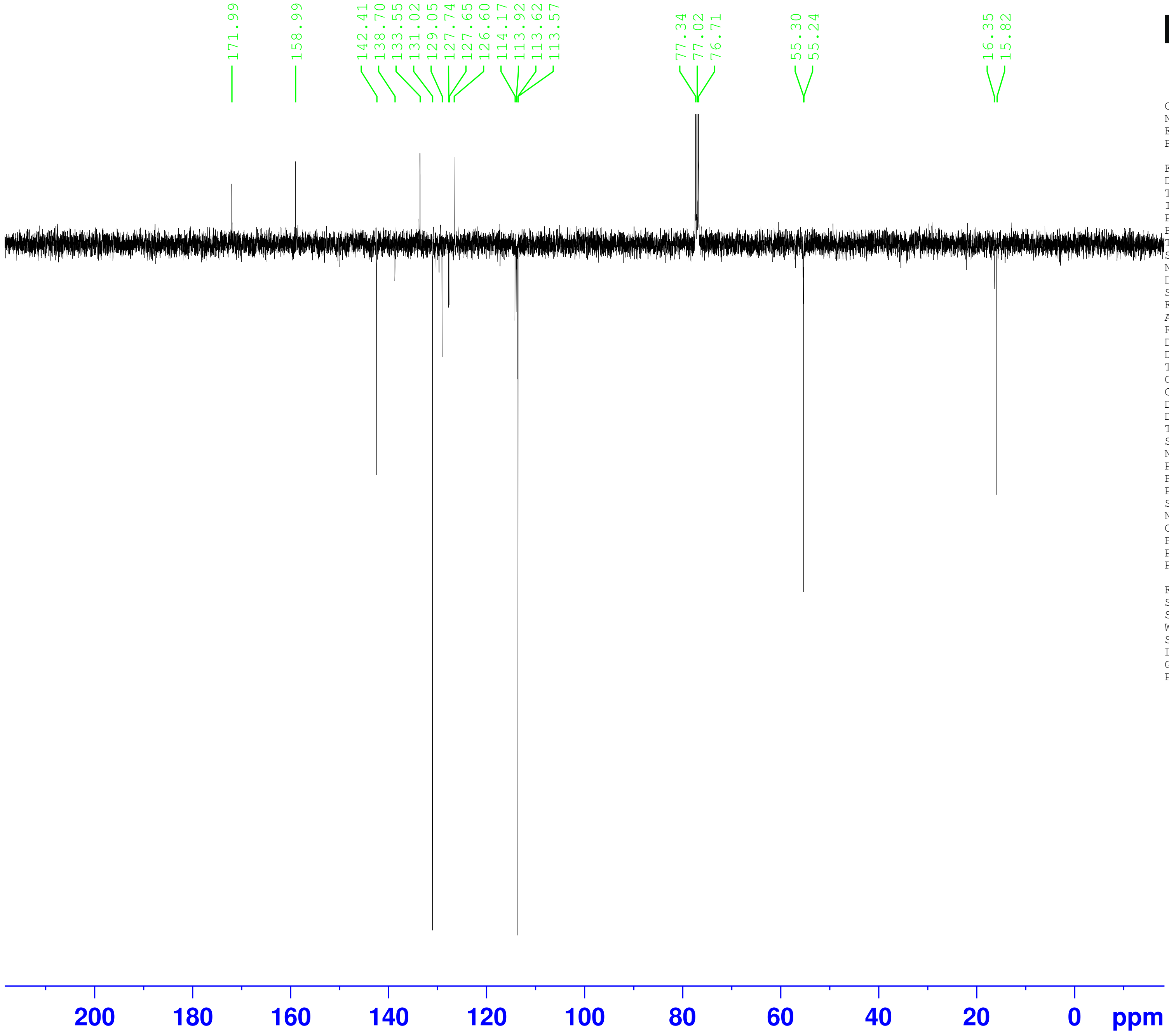

BRUKER

```
Current Data Parameters
NAME         EM04-133-crd
EXPNO                   2
PROCNO                  1

F2 - Acquisition Parameters
Date_            20230718
Time                20.53 h
INSTRUM   Avance Neo 400 U of DE Brown Lab
PROBHD    Z824801_0171 (
PULPROG              jmod
TD                  65536
SOLVENT             CDC13
NS                   1024
DS                      4
SWH             23809.524 Hz
FIDRES           0.726609 Hz
AQ              1.3762560 sec
RG                    101
DW                 21.000 usec
DE                   6.50 usec
TE                  298.0 K
CNST2         145.0000000
CNST11          1.0000000
D1             5.00000000 sec
D20            0.00689655 sec
TD0                     1
SFO1          100.6228298 MHz
NUC1                  13C
P1                  10.00 usec
P2                  20.00 usec
PLW1          44.54999924 W
SFO2          400.1316005 MHz
NUC2                   1H
CPDPRG[2          waltz65
PCPD2               90.00 usec
PLW2          16.44099998 W
PLW12          0.20298000 W

F2 - Processing parameters
SI                  32768
SF            100.6127685 MHz
WDW                    EM
SSB                     0
LB                   1.00 Hz
GB                      0
PC                   1.40
```
